# A mass spectrometry database for the identification of marine animal saponin-related metabolites

**DOI:** 10.1007/s00216-024-05586-1

**Published:** 2024-10-10

**Authors:** Stuart J. Smith, Scott F. Cummins, Cherie A. Motti, Tianfang Wang

**Affiliations:** 1 Centre for Bioinnovation, University of the Sunshine Coast, Sippy Downs, QLD 4556 Australia; 2 School of Science, Technology and Engineering, University of the Sunshine Coast, Sippy Downs, QLD 4556 Australia; 3 Australian Institute of Marine Science (AIMS), Cape Ferguson, Townsville, QLD 4810 Australia

**Keywords:** Database, Invertebrate, Marine, Mass spectrometry, Saponin, Secondary

## Abstract

Saponins encompass a diverse group of naturally occurring glycoside molecules exhibiting amphiphilic properties and a broad range of biological activities. There is a resurgence of interest in those saponins produced by marine organisms based on their potential therapeutic benefits, application in food products and most recently their potential involvement in intra- and inter-species chemical communication. The continual advancements in liquid chromatography techniques and mass spectrometry technologies have allowed for greater detection rates, as well as improved isolation and elucidation of saponins. These factors have significantly contributed to the expansion in the catalogue of known saponin structures isolated from marine invertebrates; however, there currently exists no specific chemical library resource to accelerate the discovery process. In this study, a Marine Animal Saponin Database (MASD v1.0) has been developed to serve as a valuable chemical repository for known marine saponin-related data, including chemical formula, molecular mass and biological origin of nearly 1000 secondary metabolites associated with saponins produced by marine invertebrates. We demonstrate its application with an exemplar asteroid extract (*Acanthaster* cf. *solaris*, also known as crown-of-thorns starfish; COTS), identifying saponins from the MASD v1.0 that have been previously reported from COTS, as well as 21 saponins isolated from multiple other related asteroid species. This database will help facilitate future research endeavours, aiding researchers in exploring the vast chemical diversity of saponins produced by marine organisms and providing ecological insights, and the realisation of their potential for various applications, including as pharmaceuticals.

**Graphical Abstract:**

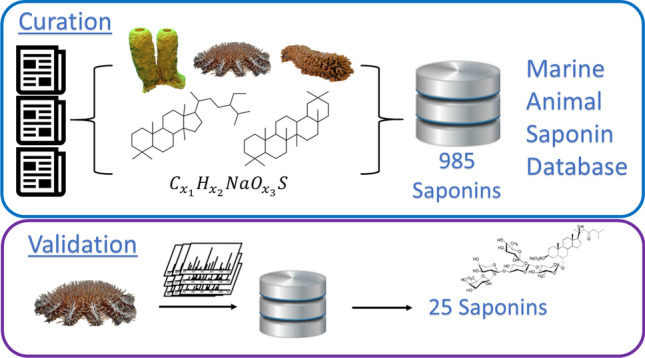

**Supplementary Information:**

The online version contains supplementary material available at 10.1007/s00216-024-05586-1.

## Introduction

Saponins encompass a class of naturally occurring amphiphilic glycosides that are biosynthesised by specific plants, bacteria and select marine invertebrates [1–4]. As secondary metabolites, they have garnered significant attention over the past five decades, in particular in the natural product discovery field [4]. This exploration has led to notable discoveries and insights into their applications, and as research delves deeper into the bioactive potential of saponins, new opportunities continue to be uncovered in areas such as pharmaceuticals, biotechnology and ecological studies.

Numerous well-characterised saponins, predominantly derived from plants, exhibit versatile properties with applications across various industries. Their surfactant properties make them valuable in detergents and cleaning products, while their potential pharmaceutical benefits include anti-inflammatory and immune-modulating properties [5, 6]. Some saponins serve as adjuvants in vaccines, enhancing immune responses [7, 8]. In the cosmetic and food industries, they are utilised as foaming agents, emulsifiers and stabilisers [9, 10]. They also play roles in agriculture as natural pesticides [11] and fisheries for controlling fish parasites [12]. Additionally, they have application in various industrial processes, including ore flotation [13] and wastewater treatment [14], as well as an assay tool in laboratory studies testing for haemolytic activity [15]. The versatility of saponins underscores their significance in a range of fields, although considerations of toxicity and safety remain essential [16, 17].

Plants are renowned for their saponin production [18, 19], however, within the kingdom Animalia, are produced by only three taxonomic classes: the echinoderms of classes Holothuroidea and Asteroidea, as well as Demospongiae sponges of class Porifera [20–22]. Each of these three classes has evolved independent biosynthetic pathways, resulting in distinct saponin structures, primarily categorised based upon the steroid or triterpenoid scaffold of the sapogenin (i.e. the hydrophobic aglycone formed through enzymatic cyclisation of lipids [23–25]), having equally distinct bioactivities [26]. This structural classification aids in understanding not only the structural diversity of animal-derived saponins, but also acts as a chemotaxonomic tool [27–30].

**Fig. 1:**
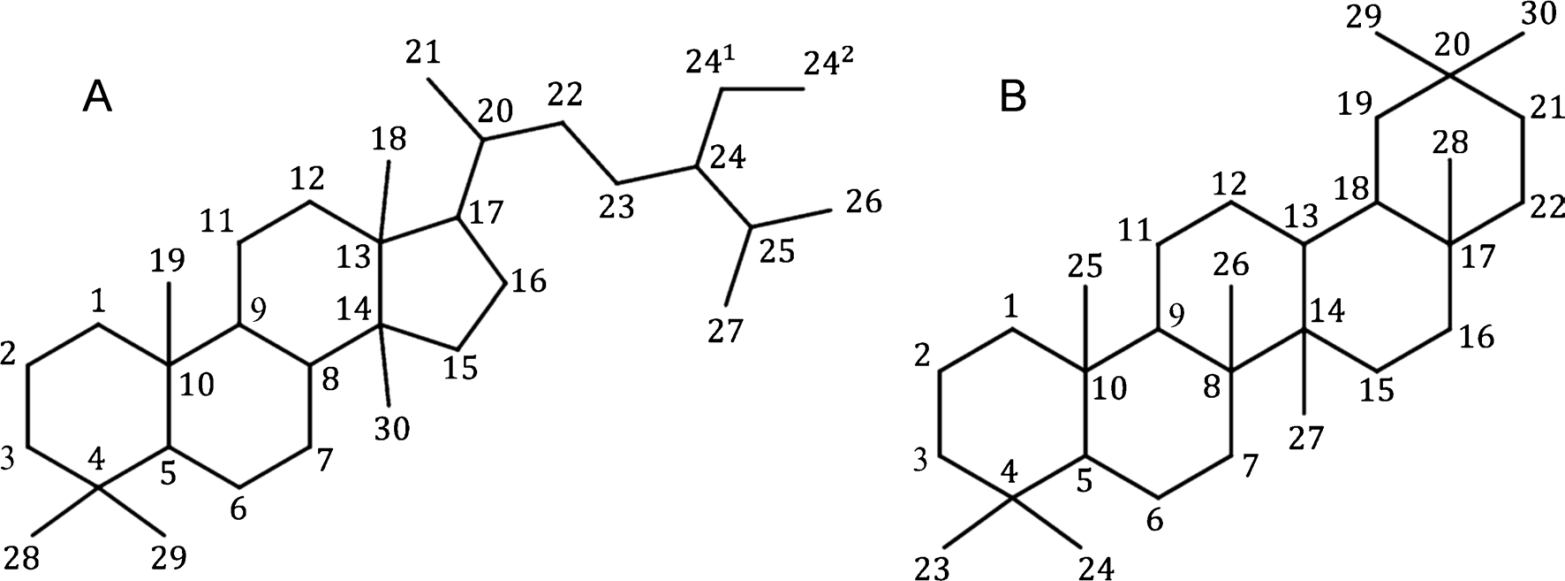
Carbon scaffold showing carbon numbering system of **A** steroid and **B** terpenoid sapogenins

Figure 1 shows the carbon scaffold of the two sapogenin classes. One or more oligosaccharide moieties are attached via either ether or ester glycosidic linkages at positions that typically harbour hydroxyl functionality, i.e. at positions C3, C6 or C20 in both sapogenins, with additional possibilities at C26 and C28 on terpenoid sapogenins [1, 4, 25, 31].

The composition of the oligosaccharide moieties varies. They are typically composed of chained monosaccharides, such as hexose (glucose, Glc; galactose, Gal; mannopyranose, Man), 6-deoxyhexose (rhamnose, Rha; fucose, Fuc; quinovose, Qui), pentose (arabinose, Ara; xylose, Xyl; ribose, Rib; apiose, Api) and uronic acid (glucuronic acid, GlcA; galacturonic acid, GalA) [32, 33]. This structural diversity within saponins contributes to the wide array of bioactive properties exhibited by the saponins and underscores their significance in various fields of research and application [26, 34].

Liquid chromatography coupled with mass spectrometry (LC–MS) serves as a robust analytical tool that enables the rapid identification and characterisation of individual small molecules within complex crude extracts and is particularly well-suited to investigate saponins [24, 35–38]. In untargeted LC–MS analysis, this method produces a vast amount of data from crude extracts, underscoring the need for extensive compound libraries against which MS data can be screened. Matching observed mass-to-charge ratio (*m*/*z*) values with entries in a relevant database facilitates the referencing of known compounds, aiding in the rapid identification of structurally elucidated saponins from any sample [39]. Notably, there remains a critical gap in this domain. Recently, an MS database specifically focussing on plant saponins has been published; however, no such centralised database exists that is tailored to saponins produced by marine invertebrate animals [40]. Establishing such a resource would significantly enhance the efficiency and depth of saponin identification and characterisation within this unique context.

In this study, we created a database dedicated to the elucidation of saponins derived from marine invertebrates, with inbuilt capability for future expansion as new information becomes available. Encapsulating saponin-related knowledge through the systematic review and collation of published structures into a tailored database will provide a valuable resource that can expedite the screening of known marine-animal-derived saponins as well as the discovery and accurate identification of new marine saponins thereby contributing to a deeper understanding of the structural features and species specificity of marine saponins. Importantly, to validate the usefulness of MASD v1.0, we applied it to investigate the largely understudied saponins produced by *Acanthaster* cf. *solaris* (also known as crown-of-thorns starfish; COTS), an asteroid which has found recent infamy due to its dire impact on coral reefs [41–43].

## Materials and methods

### Curation of the Marine Animal Saponin Database

**Fig. 2:**
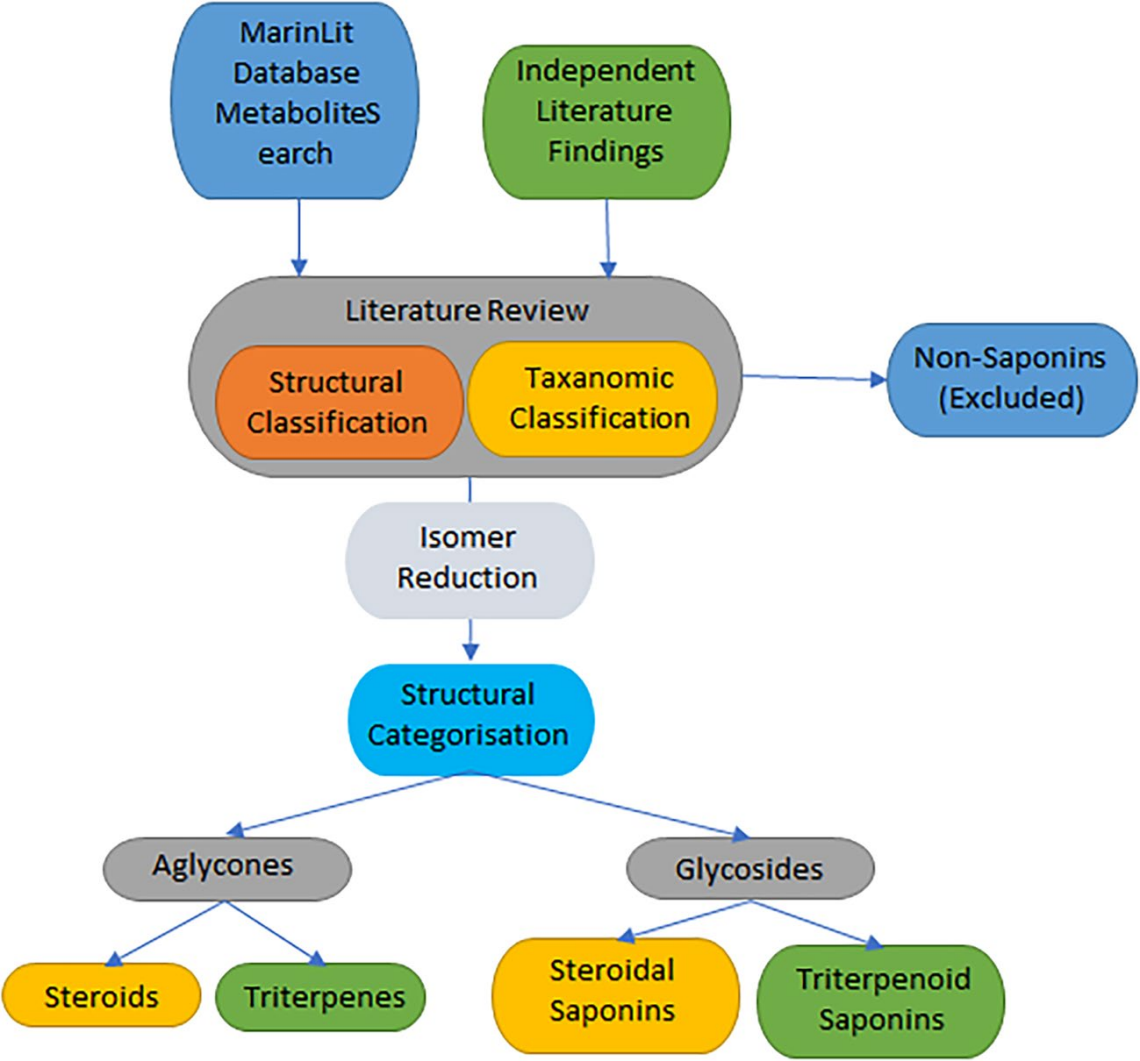
Flow diagram illustrating the procedure of curating the marine animal saponin database (MASD v1.0) from resources

The establishment of a Marine Animal Saponin Database version 1.0 (MASD v1.0) utilised a systematic workflow, as illustrated in Fig. 2. Initially, chemical and structural data was extracted from the MarinLit database (https://marinlit.rsc.org/) based on key search criteria: taxa = Animalia, Asteroidea, Holothuroidea or Demospongiae, or common name/term = saponin or sapogenin or aglycone, molecular mass ≤ 2150 Da. Outputs (structural, taxonomic and bibliographic) were curated based on specific criteria, including chemical formula and molecular mass (within the range from 330 to 2150 Da), structural and functional elements and the genus and species from which they were isolated (specifically within the classes Asteroidea, Holothuroidea or Demospongiae). The structural categorisation process involved a deep dive analysis of the chemical structures reported in the publications sourced from the MarinLit database. Metabolites with structures representing saponins were systematically extracted. Further, additional publications that focused on elucidating saponin-related metabolites were categorised, encompassing both complete saponin structures and sapogenins.

A review of cited references in the amassed publications, as well as keyword searching in Web of Science (parameters = “Demospongiae”, “Holothuroidea”, “Asteroidea” and “saponin”; and parameters = “steroidal glycosides” or “triterpenoid glycosides”), was undertaken to identify any other relevant web-based publications (Fig. 2). In-depth analysis sorted entries into steroid or triterpenoid structural categories, primarily based on the number of complete carbon rings present within the aglycone. Specifically, structures with a core consisting of three 6-member rings and one 5-member ring would be classed as steroidal (Fig. 1A), while those having four 6-member rings were classified as triterpenoid (Fig. 1B). Further categorisation based on the presence or absence of a glycoside chain was performed, resulting in the final classification of molecules. Notably, compounds exhibiting structural elements unrelated to saponins were meticulously identified and removed from the dataset, ensuring precision and accuracy in the subsequent analyses.

In the process of structural classification, we identified multiple instances where two or more confirmed saponin structures shared identical chemical formulas. To enhance the coherence of our database, for this draft version such entries, if originating from the same marine animal source, they were merged into a singular entity, as the structural isomers would be indistinguishable via MS^1^ analysis; all associated compound names and sources were denoted appropriately. Beyond structural classification, we placed significant emphasis on capturing crucial information regarding the marine animal species from which the saponins were originally elucidated. This documentation ensures that our database not only serves as a comprehensive resource for structural information but also provides valuable insights into the biodiversity and ecological contexts associated with these marine-derived saponins.

Having accomplished the structural categorisation, elucidation species determination and confirmation of chemical information for each individual data entry, the dataset was systematically organised into an Excel spreadsheet for enhanced usability. The spreadsheet comprises essential elements, including a unique ID number assigned to each entry which comprises the following information: molecular mass, molecule name, sourced species, taxonomic class of the sourced species, molecular formula, structural category, molecular mass, elucidation publication reference and the year of publication. These comprehensive elements provide a robust framework for efficiently navigating and manipulating the database contents as needed, ensuring accessibility and ease of use.

### Utilisation of MASD v1.0 for mass spectrometry data from the crown-of-thorns starfish

The completed MASD v1.0 filtering function was utilised to extract entries labelled “Asteroidea”, segregating entries pertaining to saponins originating from species within the class Asteroidea (starfish). These refined entries were then compiled into a text (txt) file, capturing essential information like “Chemical names” and “Chemical formula”. Subsequently, this txt file was integrated into the SCIEX OS Analytics control terminal, becoming a pivotal component of an innovative data analytics procedure. This integration facilitated a focused analysis of MS data files, specifically derived from the exploration of extracts of *Acanthaster* cf. *solaris*.

### Animal collection and tissue extraction

**Fig. 3:**
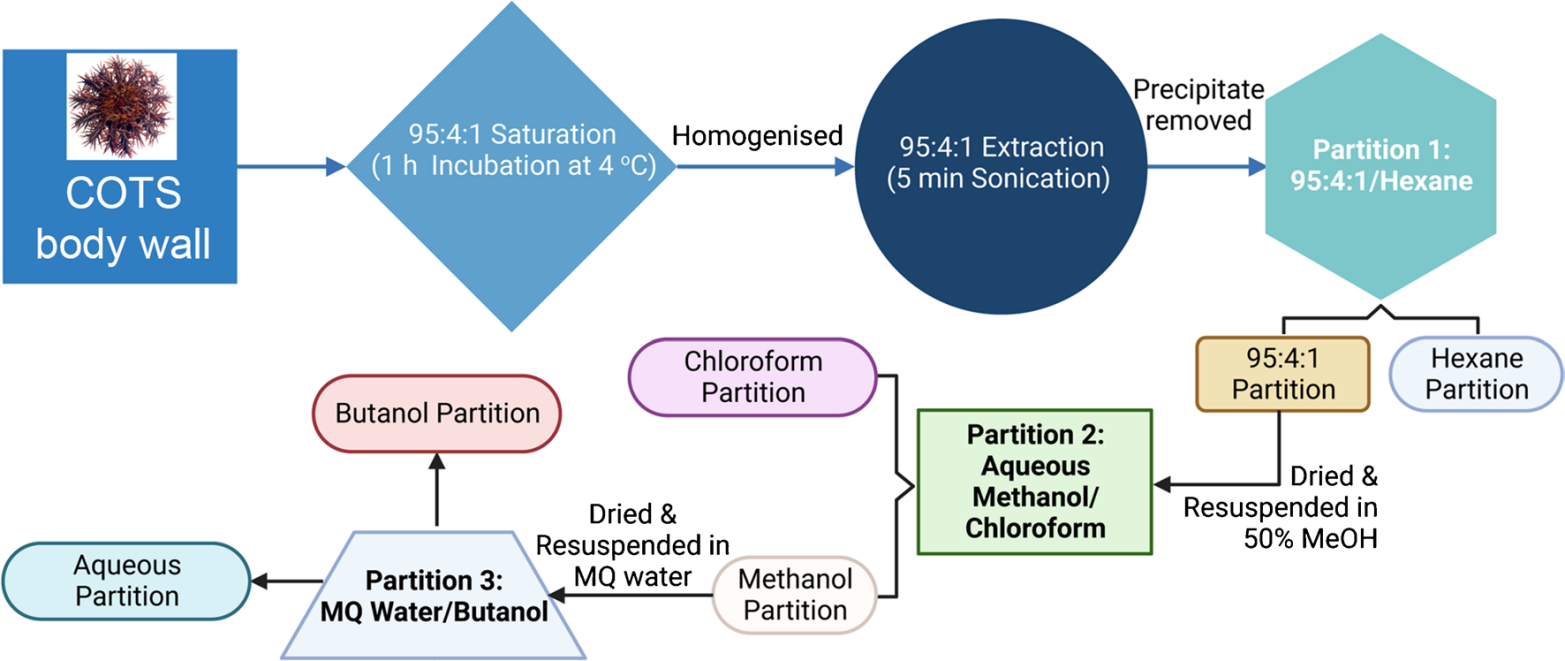
Workflow for the isolation of saponins from the body wall tissues of crown-of-thorns starfish (*n* = 4)

The overall experimental design for the isolation of saponins from adult COTS tissue is outlined in Fig. 3. Ethics approvals are not required on echinoderms. Four adult COTS (sex undetermined) were collected by the Australian Marine Park Tourism Operator (AMPTO) divers from the Great Barrier Reef (GBR), off the coast of Cairns. They were transported to the University of the Sunshine Coast (Sippy Downs, QLD) aquaculture facility where they were maintained in a communal flow-through tank. Four arms were amputated at the central disk terminus of each individual starfish. Each arm was further processed by removing the prominent spines, isolating the body wall from the calcareous skeleton (totalling approximately 11 g) followed by immersion in 40 mL of a 95% methanol/4% water/1% acetic acid solution (95:4:1). After incubation at 4 °C for 1 h, tissue was homogenised (Cole-Parmer LabGEN 7) on ice, reducing it to a fine slurry. Subsequently, the mixture was sonicated in a Powersonic 603 (Kleentek) set at 40 kHz (Volume 3) for 5 min. The sample underwent gravity filtration over the Whatman Grade 2 Filter Paper, and the filtrate was stored at − 20 °C to promote salt precipitation [44]. After overnight incubation, the supernatant was decanted from the precipitate, and the solids disposed. The supernatant (~ 40 mL of 95:4:1 solution) was solvent-partitioned (i.e. in a 100 mL separating funnel) with 40 mL of *n*-hexane. The mixture was inverted repeatedly for ~ 1 min and then allowed to settle for 5–10 min, after which the two layers were collected and each dried under centrifugal vacuum (Savant SpeedVac).

The dried polar extract was resuspended in 40 mL of 50% aqueous methanol and solvent-partitioned with 40 mL chloroform (as above). Both layers were collected and dried (as above). Finally, the dried aqueous methanol extract was resuspended in 40 mL of Milli-Q water and solvent-partitioned with 40 mL of *n*-butanol. After partitioning, both the aqueous and butanol layers were collected and dried under rotary evaporation (Genevac EZ-2 personal evaporator, Sciteck, Australia).

### Saponin identification using mHPLC-QToF-MS analysis

The butanol extract was subjected to comprehensive analysis using ExionLC liquid chromatography system (AB SCIEX, Concord, Canada) coupled to a X500R QToF mass spectrometer (AB SCIEX, Concord, Canada) equipped with an electrospray ion source (LC–MS). A 10 mL aliquot was injected onto a 150 × 4.6 mm bioZen 3.6 μm Intact C_8_ mHPLC column (Phenomenex, Sydney, Australia), fitted with a SecurityGuard column with a flow rate set to 0.4 mL/min. Gradient elution was performed using solvent A (0.1% aqueous formic acid) and solvent B (95% acetonitrile/5% Milli-Q water/0.1% aqueous formic acid) and the elution gradient optimised for peak separation as depicted in Table 1. Between samples, solvent B was held at 100% for 1 min to wash the column then returned to 100% solvent A for equilibration prior to the next injection. The ion spray voltage was set to 5500 V, declustering potential (DP) 100 V, curtain gas flow 30, ion source gas 1 (GS1) 40, ion source gas 2 (GS2) 50 and spray temperature at 500 °C. The mass spectrometer acquired MS data in an Information Dependant Acquisition, IDA mode. Full scan ToF–MS data was acquired over the mass range 350–1500 m*/z*.

**Table 1:** Solvent gradient for liquid chromatography

Time (min)	Solvent A (%)	Solvent B (%)
0	100	0
2	95	5
6	91	9
6.5	75	25
16	55	45
24	53	47
28	49	51
30	43	57
32	40	60
36	20	80
38	0	100
40	0	100

### Data analysis

Utilising SCIEX OS Analytics software, data processing involved the application of the MASD v1.0, restricting the database selection to “Asteroid” and “Steroidal Saponins”. Peak identification and calculation were performed using the full width at half maximum (FWHM) for each detected peak. Specifically, peaks within half the peak height were included in the analysis to ensure accurate representation of the peak shape. Peak integration parameters included a minimum peak width and height set to 3 and 50.00 points, respectively. A signal-to-noise threshold of 3 and an XIC width of 0.02 Da were implemented. Acceptance criteria encompassed a lower limit of integration quality at 0.800, an asymmetry factor between 0.500 and 20.000 and the total width having an interval of 0.250 to 0.500. Qualitative rules were established, requiring a mass error less than 2 ppm and an isotope ratio less than 5%.

## Results

### Overview of the MASD v1.0

**Fig. 4:**
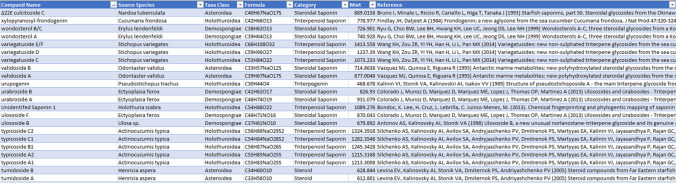
The layout of MASD v1.0. The interface includes ID#, compound name, animal source species, animal taxa class, chemical formula, structural category and molecular weight. It allows users to perform saponin searches by applying filters to different columns. This feature facilitates efficient identification and analysis of saponins based on various parameters

**Fig. 5:**
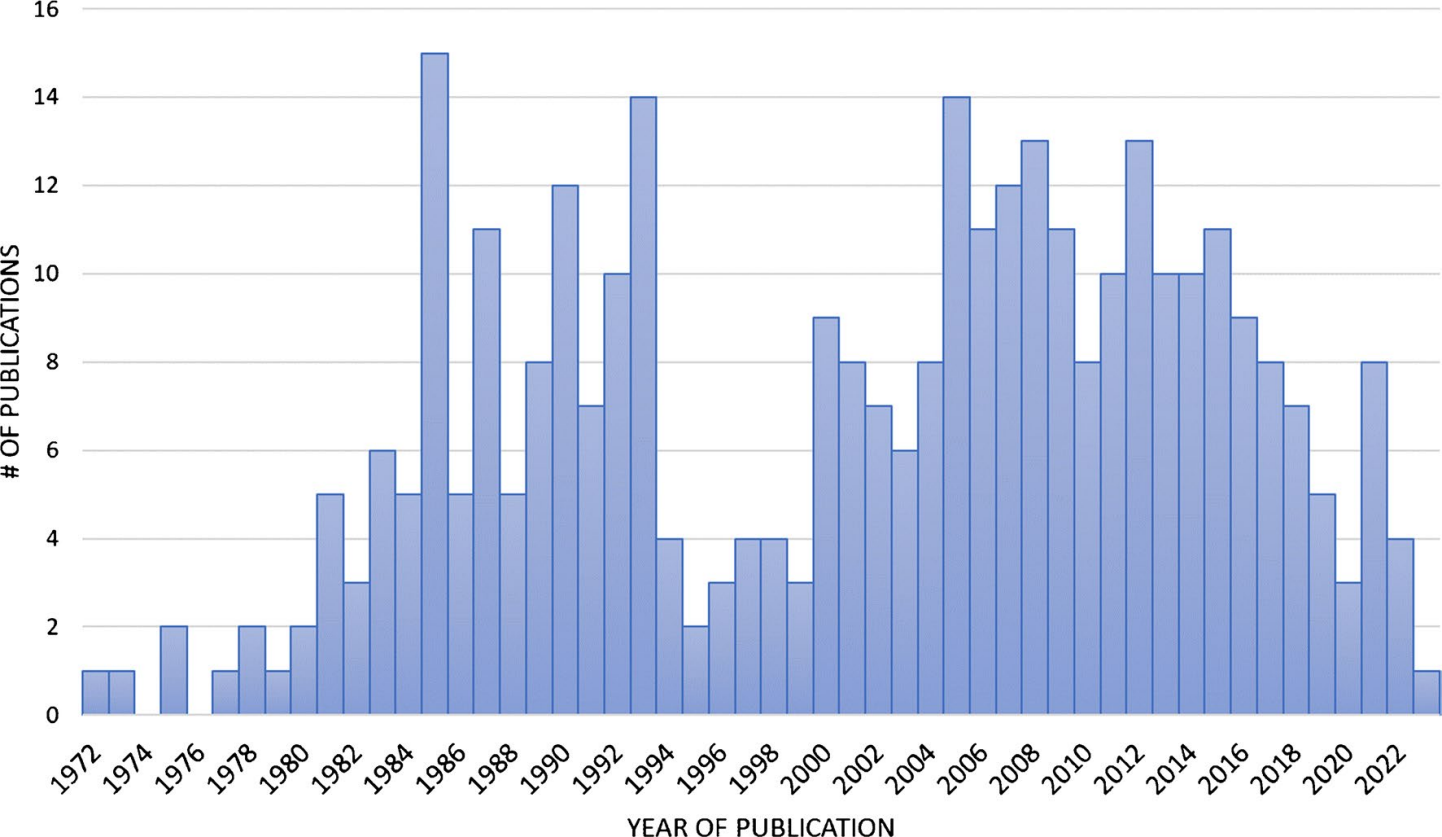
Year of publication for journal articles utilised in the build of MASD v1.0 (see Electronic Supplementary Material File S1 for the full list of publications)

A total of 386 publications reporting on marine saponins were recovered from the MarinLit database and an additional 29 from cited sources. The final number of saponins in the MASD v1.0 was reduced from 1463 to 985, curated from 346 chemical discovery publications (Electronic Supplementary Material File S2). Each entry within the database is linked to the publication from which it originated, which serves to inform the year of discovery, elucidated chemical structure, structural classification and species of marine animal from which the saponin was isolated (Fig. 4). The temporal distribution of publications contributing to this database **(**Fig. 5**)** revealed two notable surges in research output related to the isolation and elucidation of saponin-related natural products from marine animal sources. During the first decade, around 15 papers were published that primarily described the isolation of individual or paired saponin-related structures from both holothuroid and asteroid species; most of these featured studies were conducted as pharmacological investigations of toxic extracts at Osaka University (Japan).

### Classification of chemical structures based on taxonomic class and species

The MASD v1.0 database employed three primary search categories:

1. Chemical structure: This category allows users to search for saponins based on their chemical structures, making it convenient for researchers interested in specific types of saponins.
2. Animal taxonomic class: Users can search for saponins based on the taxonomic class of the marine animal from which they were isolated. This categorisation aids in exploring the diversity of saponins within a specific marine class.
3. Animal species: This category permits searching based on the specific species. This is particularly useful for researchers interested in changes in saponin profiles that may provide insight into ecological function.

**Fig. 6:**
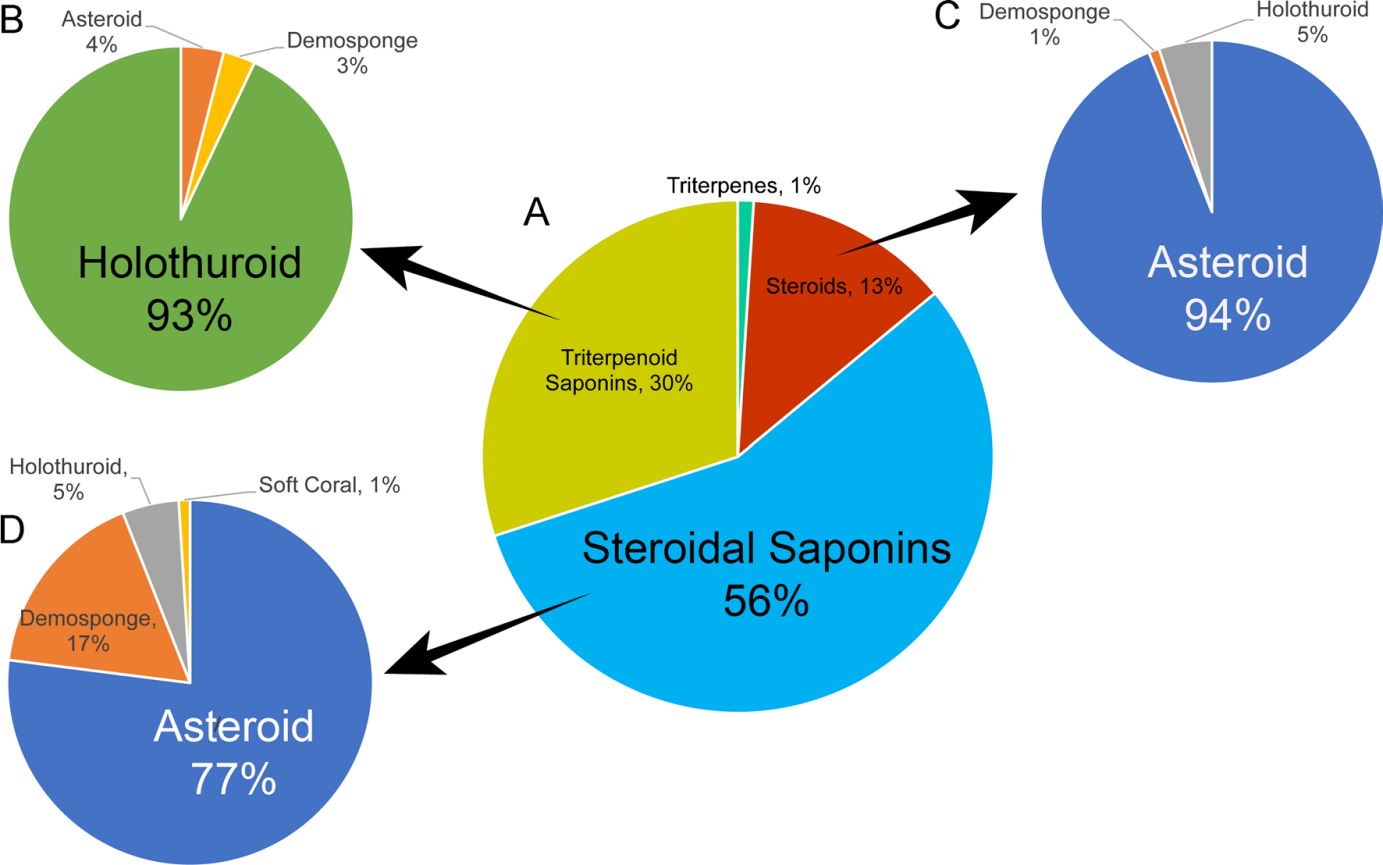
Classification of saponin-related compounds collated in the Marine Animal Saponin Database (MASD) V1.0 and the source taxonomic class. **A** Structural composition of MASD v1.0 entries. **B** Taxonomic class distribution of triterpenoid saponin entries. **C** Taxonomic class distribution of sapogenin entries. **D** Taxonomic class distribution of steroidal saponin entries

A primary focus was placed on the classification of the aglycone, the identifiable core structure of the saponin molecule. A substantial majority of entries in MASD v1.0, accounting for 86%, fall into the category of fully glycosylated triterpenoid or steroidal saponins (Fig. 6A), with the remaining portion, comprising the non-glycosylated version of both variants. Of the fully glycosylated saponin entries, 30% were categorised as triterpenoid saponins, with 93% originating from holothuroid species (Fig. 6B) and limited entries being derived from asteroid or demosponge species. The sapogenin portion of database entries, however, did not reflect this composition with only 5 entries categorised as triterpene sapogenins among the 14% of total molecules identified as sapogenin (Fig. 6A); almost all (94%) sapogenin entries were found to be steroidal in structure, originating primarily from asteroid sources (Fig. 6C). Many of these steroid entries were identified as the hydrolysed aglycone form of glycosylated steroidal saponins, which comprised approximately two-thirds of the database, predominantly reported from asteroid species sources (Fig. 6D).

Entries from specimens of the Demospongiae class constituted approximately 9.5% of the total database, comprising a mix of triterpenoid and steroidal structures. Notably, 1% of steroidal saponin entries originated from the Octocorallia class of soft corals (Cnidaria), showcasing aglycone structures which approximate the steroidal configuration typically found in Demosponge steroidal saponins.

**Fig. 7:**
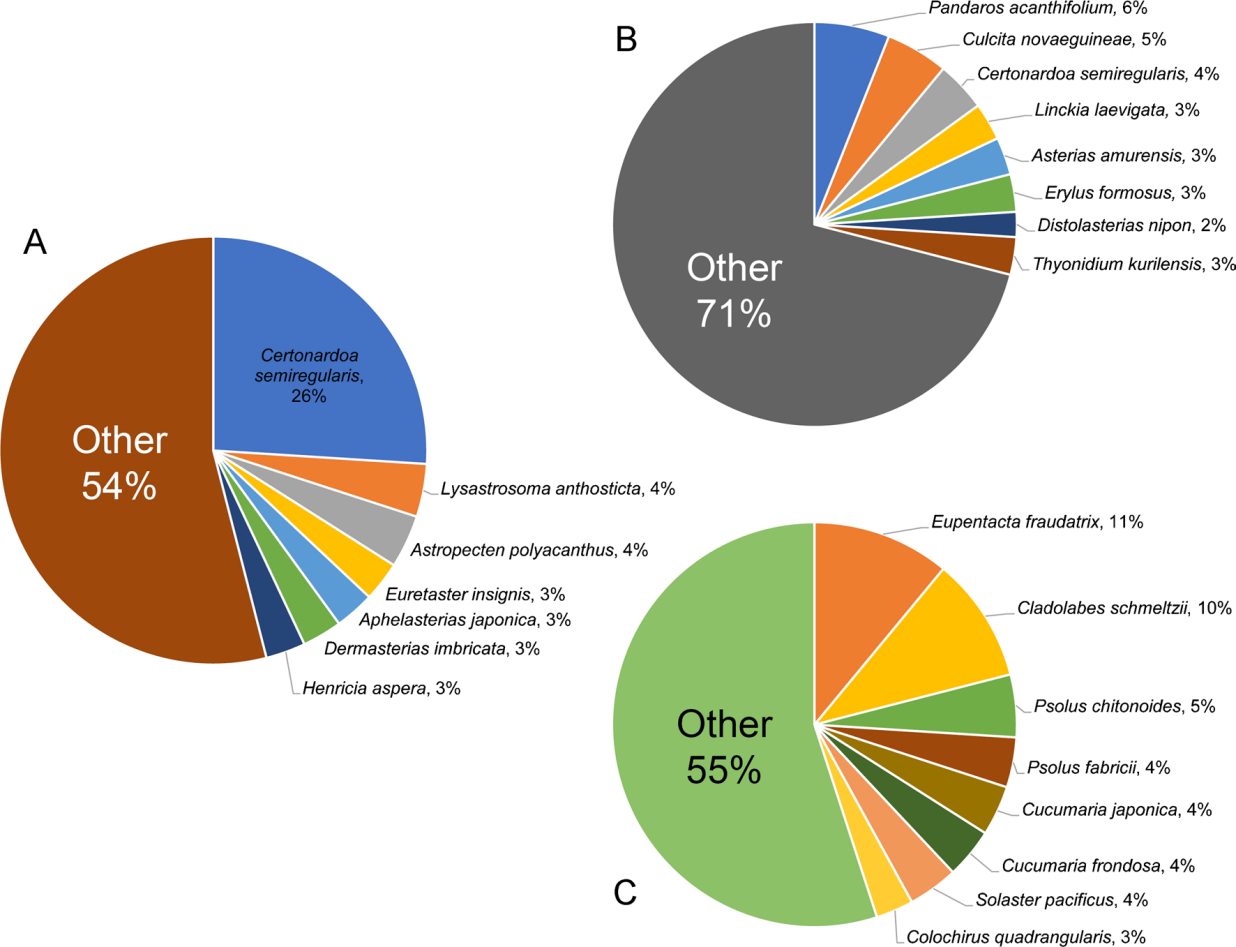
Relative distribution of source species containing the three saponin types: **A** aglycone molecules (steroidal and triterpenoid), **B** steroidal and **C** triterpenoid

Species classification categorised saponins and sapogenins based on the specific marine animal species from which the metabolite was originally isolated and elucidated. This classification system allows for improved identification of structures shared between related species. The sapogenins portion of the database (Fig. 7A) is predominantly comprised of molecules isolated from asteroid species (see Fig. 6) with 26% of all elucidated sapogenins isolated from the asteroid *Certonardoa semiregularis* in three studies published between 2002 and 2004 (Electronic Supplementary Material File S1). The other highest contributors included six species of asteroids, each contributing several steroidal sapogenins, accounting for a 3 to 4% share of the total sapogenin catalogue in this database. The elucidation of full glycosylated saponins (Fig. 7B, C) showed a varied distribution of source species, with steroidal saponins originating primarily from asteroid and demosponge species. Notably, a single holothuroid species, *Thyonidium kurilensis* (Fig. 7B), was among the top eight species contributing to saponin entries, which contrasts with the distribution of the triterpenoid saponins (Fig. 7C), where holothuroid species dominate, representing all but one of the top eight contributors, the exception being the asteroid species, *Solaster pacificus*.

## Identification of saponins in COTS body wall utilising MASD v1.0

MS analysis of extracts derived from COTS body wall yielded 512 small molecule mass peaks meeting the specified criteria. Utilising the MASD v1.0 database, 25 full steroidal saponin matches were identified within confidence parameters. Among those, four saponins had been previously reported from the *Acanthaster* species complex (Table 2). Additionally, 21 steroidal saponin structures were identified, originating from asteroid species outside the *Acanthasteridae* family (Table 3). The number of hydroxyl functional groups on the saponins identified varied from 5 to 14, indicating a range in the hydrophilicity of these compounds. Of the 25 compounds identified by the MASD v1.0 as steroidal saponins, six mass peaks correlated to two possible saponin entries, due to several possessing the same exact molecular mass. This indicates that while the chemical formula identified the molecules as saponins, the database was unable to determine their exact structural identities. This suggested the presence of structural isomers, where two compounds share the same elemental composition but differ in their structural configurations.

**Table 2:**
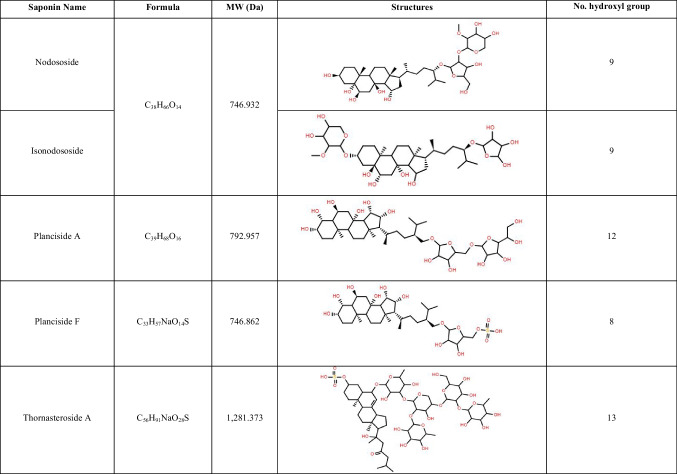
List of steroidal saponins identified in *Acanthaster* body wall and matching compounds elucidated from members of the *Acanthasteridae* family using MASD v1.0

**Table 3:**
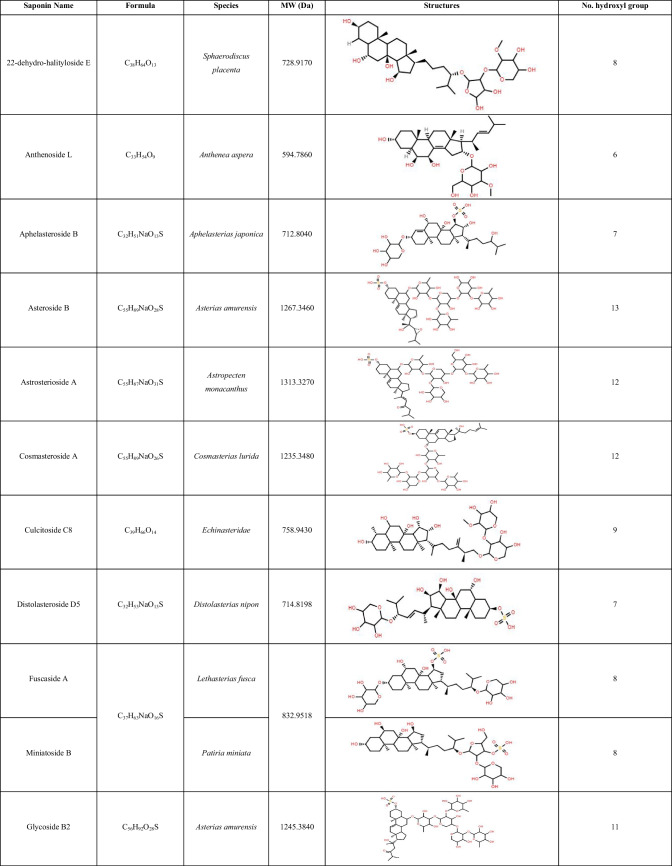

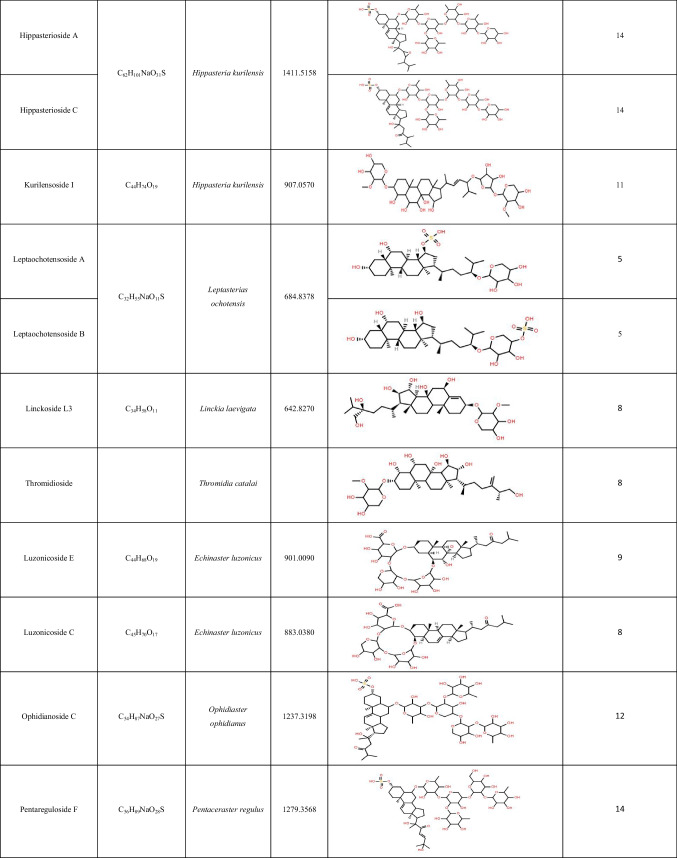

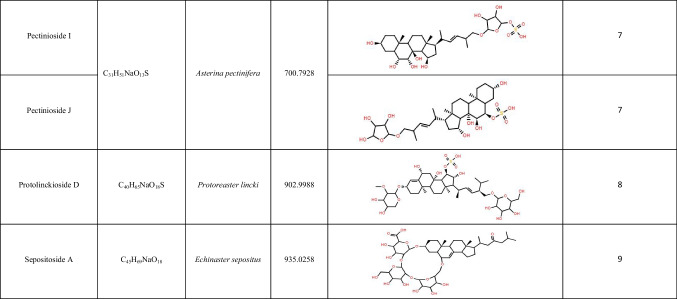
List of steroidal saponins identified in *Acanthaster* body wall and matching compounds elucidated from members of the *Acanthasteridae* family using MASD v1.0

## Discussion

Saponins have been identified in a variety of different marine invertebrate species, having a range of chemical structures and biological activities. With the isolation and elucidation of marine saponins increasing, facilitated by advanced LC–MS techniques, the timely development of the MASD v1.0 provides a means to explore saponin diversity by rapidly screening MS data against documented chemical and taxonomic information to identify known saponins and identify putative novel molecules including new saponins [39, 44–46].

### The MASD v1.0: a comprehensive saponin database

Our interrogation of the MarinLit database and the web-based literature led to the curation of 985 metabolites, incorporating 244 references that provide data verification of the structure and enable searching at the class and species taxonomic levels to refine the output. This dual approach to extract the relevant information ensured a robust and extensive collection of chemical data, capturing a wide array of saponin-related metabolites from marine invertebrate sources. Additionally, the use of multiple references for structure verification, i.e. where a saponin is reported from multiple species, improved the reliability and relevance of the curated dataset.

Since the initial identification of Asterosaponin A in 1960 [29, 47], there have been two periods of intense research activity culminating in the publication of saponin-related research papers, attributable to several factors. The first spike, from 1984 to 1993, was primarily due to pharmacological investigations spurred on by recent advancements in extraction and analytical techniques, making it easier for researchers to identify and characterise the saponin structures. These advancements included improved LC–MS instrumentation and techniques, and the increased prevalence of structurally deterministic technologies such as ^13^C nuclear magnetic resonance (NMR) and 2D NMR over proton NMR (^1^H NMR) [48, 49]. Additionally, increased interest in marine natural products for pharmaceutical and industrial applications spurred more investigations, with early saponin bioactivity publications appearing between 1978 and 1985 [22, 50, 51]. The second spike, from 2000 to 2022, can be directly attributed to a growing awareness of the therapeutic potential of marine-derived compounds, including saponins; this period saw a dominance of bioactivity-related publications in the MASD v1.0 (see Electronic Supplementary Material File S1) entries. The shift from basic identification and characterisation to a focus on bioactivity and therapeutic potential highlights the evolving nature of saponin research. The sustained interest in saponins, as evidenced by the continuous research output, confirms the ongoing importance of these compounds [52, 53].

### Structural trends

While saponins exhibit distinctive structural elements among different taxonomic classes of marine invertebrates, variation in structure at the species level is still observed [54]. This variability underscores the complexity of saponin chemistry within marine invertebrates. Taxonomic classification of the species from which the saponin was initially isolated and then re-isolated is crucial. The MASD v1.0 has captured many publications reporting the presence of specific saponins previously identified in species of other genera or families within the same taxonomic class [55, 56], quickly identifying the potential structural similarities or shared biosynthetic pathways among related species, and offers a rapid means of chemotaxonomic classification [26].

Alongside saponin structures, MASD v1.0 includes the classification of the aglycone, the core structure of the saponin molecule, due to its central role in saponin chemistry and functionality. In many instances, the sapogenin has been isolated alongside the complete saponin structure, either as a co-occurring biosynthetic precursor or as a hydrolysed product resulting from the isolation process. The predominance of steroidal non-glycosylated structures within the database, compared to the relatively few examples of triterpene sapogenins, underscores the prevalence of steroidal aglycone co-occurrence in saponin-related articles, particularly with respect to asteroid species (see Fig. 4). A notable example is the diversity of steroidal aglycones elucidated from *Certonardoa semiregularis* [57–59], emphasised by the distribution of source species in the MASD v1.0 (see Fig. 5). These findings provide valuable insights into saponin biosynthesis and their chemical transformations and support lab-based synthesis for industry, medicinal and scientific application [54, 60–62].

A comparable resource to the MASD v1.0 is the Saponin Mass Spectrometry Database (SMSD) [40], which focuses solely on plant saponins. Similar to this study, the SMSD primarily utilised an established metabolomics database, i.e. PubMed, to identify 4196 metabolites from matched keywords. Plant saponin research is very mature, and with 214 saponin standards available, data pertaining to MS fragmentation behaviour for multiple structural variations is available within the SMSD [40]. Relatively, the paucity of high-purity invertebrate saponin standards currently prevents the MASD v1.0 from providing verified fragmentation behavioural data.

### MASD v1.0 provides an in-depth analysis of COTS saponins

Interrogation of the MASD v1.0, identified 13 steroidal saponins originally elucidated from COTS isolations, reported in eight publications, between 1978 and 2019 [22, 56, 60, 63–66]. Here, the saponin composition of a semi-crude COTS body wall extract was profiled by screening the LC–MS data against the MASD v1.0, allowing for rapid analysis and identification of a total of 25 saponins (see Tables 2 and 3) [56, 65]. Four of the saponins identified matched to saponins originally elucidated from COTS isolations, while an additional 21 were identified as saponins elucidated from other asteroid species isolations, once more indicating shared biosynthetic pathways between asteroid species.

In addition, when comparing the data to other studies reporting on saponins from various COTS tissues, the majority of identified structures either matched directly or have structural similarity to the reported saponins, as expected [45, 56]. Documenting the complexity of COTS saponin structural diversity is essential for evaluating their potential utility as a source of biological active chemistry. Studies investigating the bioactivity of saponins typically identify common structural elements (or scaffolds) present in the more bioactive saponin structures, for example, the presence, number or arrangement of hydroxyl groups on the aglycone. These factors can influence solubility, membrane permeability and interactions with biological targets, affording specific saponins unique pharmacological properties, such as antioxidant, antimicrobial or anticancer activities [67]. These hydroxylation patterns can reflect genetic variability, the impact of environmental factors or ecological interactions, providing valuable information for taxonomic classification and ecological studies [68, 69].

There is evidence that marine invertebrates secrete saponins into their surroundings, and that these saponins impart biological function, including deterrence against predators, antimicrobial properties and allelopathic effects on competing organisms [70–74]. An initial SAR assessment (Tables 2 and 3) indicates COTS saponins are heavily hydroxylated, yet it is unclear whether these functionalities are what impart the above effects. The elucidation of the role of these saponins in COTS biology and ecology warrants further investigation, in particular, with saponins hypothesised to be involved in chemical communication, such knowledge may inform the design and development of control strategies to modify COTS behaviours and mitigate the negative impacts of COTS outbreaks on coral reefs thereby promoting reef resilience [41, 42].

### Limitations of MASD v1.0

The MASD v1.0 currently provides only the MS^1^ profile for characterised saponins and sapogenin molecules, based on available information documented in the available academic literature. This limitation restricts the database’s specificity and identification capabilities, as it does not offer detailed data on the fragmentation patterns needed to identify the exact location of functional groups on the target molecules nor can it distinguish isomers. Consequently, while it provides a broad overview of saponin structures, it currently lacks the depth needed for comprehensive analysis. To address this limitation and enhance the database's analytical capabilities, future iterations should adopt a multi-tiered structural library that includes:

1. Fully glycosylated saponin structure: This top tier would describe the complete saponin structure, confirmed by MS and 1D and 2D NMR.
2. Sapogenin and glycoside segmentation: The second tier would provide a link to the associated (or predicted) sapogenin and glycoside components, allowing for an in-depth examination for the presence of these structural elements.
3. Tandem MS^n^ profiles: The third tier would include observed MS^n^ fragmentation patterns for specified saponin-related molecules, allowing for enhanced specificity and differentiation between structural isomers. By revealing fragmentation patterns, it would provide valuable insights into the detailed structural characteristics of saponins, aiding in their rapid identification and characterisation.

## Conclusions

The MASD v1.0 is a collation of chemical elucidation data specific to marine animal saponins, organised with the specification of key search parameters that facilitate targeted secondary metabolite analyses. The MASD v1.0 serves as a central repository for data related to animal-derived saponins and the species from which they have been isolated, effectively addressing a current knowledge gap in secondary metabolite reference tools. It is a comprehensive and organised resource, based on a methodical workflow, that facilitates the structural identification of saponins (through MS technology) from marine invertebrates and will assist in unravelling the complexity of saponin chemistry of natural products to inform on their potential application, including in drug discovery and ecological studies.

## Supplementary Information

Below is the link to the electronic supplementary material.

Supplementary file1 Electronic Supplementary Material File S1. Marine Animal Saponin Database and associated references (XLSX 118 KB)

## Declarations

### Source of biological material

The COTS used in this study were collected by divers from the Australian Marine Park Tourism Operator (AMPTO) under the GBRMPA permit G18/41587.1.

### Statement on animal welfare

As COTS are not regulated under animal welfare guidelines, no formal ethics approval was required for their collection or use in this study. However, all efforts were made to handle and manage the animals responsibly, adhering to best practices for fieldwork and specimen handling in line with environmental and conservation standards.

## References

[1] Francis G, Kerem Z, Makkar HP, Becker K. The biological action of saponins in animal systems: a review. Br J Nutr. 2002;88(6):587–605. 12493081 10.1079/BJN2002725

[2] Francis G, Kerem Z, Makkar HPS, Becker K. Reflections on ‘The biological action of saponins in animal systems: a review.’ Br J Nutr. 2022;127(7):1034–6. 34913419 10.1017/S0007114521004852

[3] Sumitha R, Banu N, Parvathi VD. Novel natural products from marine sea stars. Curr Trends in Biomed Eng Biosci. 2017;2(4):59–63.

[4] Desai SD, Desai DG, Kaur H. Saponins and their biological activities. Pharma Times. 2009;41(3):13–6.

[5] Golmohammadi MG, Banaei S, Timar M, Abedi A. Saponin protects against cyclophosphamide-induced kidney and liver damage via antioxidant and anti-inflammatory actions. Physiol Int. 2023;110(2):108–20. 37256739 10.1556/2060.2023.00190

[6] Dai Z, Zhu PF, Liu H, Li XC, Zhu YY, Liu YY, et al. Discovery of potent immune-modulating molecule taccaoside A against cancers from structures-active relationships of natural steroidal saponins. Phytomedicine. 2022;104: 154335. 35858515 10.1016/j.phymed.2022.154335

[7] Marciani DJ. Elucidating the mechanisms of action of saponin-derived adjuvants. Trends Pharmacol Sci. 2018;39(6):573–85. 29655658 10.1016/j.tips.2018.03.005

[8] Ragupathi G, Gardner JR, Livingston PO, Gin DY. Natural and synthetic saponin adjuvant QS-21 for vaccines against cancer. Expert Rev Vaccines. 2011;10(4):463–70. 21506644 10.1586/erv.11.18 PMC3658151

[9] Timilsena YP, Phosanam A, Stockmann R. Perspectives on saponins: food functionality and applications. Int J Mol Sci. 2023;24(17):13538. 37686341 10.3390/ijms241713538 PMC10487995

[10] Bezerra KGO, Rufino RD, Luna JM, Sarubbo LA. Saponins and microbial biosurfactants: potential raw materials for the formulation of cosmetics. Biotechnol Prog. 2018;34(6):1482–93. 30051974 10.1002/btpr.2682

[11] Chaieb I. Novel advances and perspectives to the use of plant saponins as pesticides. Acta Hortic. 2013;997:177–84.

[12] Wang GX, Han J, Zhao LW, Jiang DX, Liu YT, Liu XL. Anthelmintic activity of steroidal saponins from Paris polyphylla. Phytomedicine. 2010;17(14):1102–5. 20576414 10.1016/j.phymed.2010.04.012

[13] Oulkhir A, Lyamlouli K, Danouche M, Ouazzani J, Benhida R. A critical review on natural surfactants and their potential for sustainable mineral flotation. Rev Environ Sci Bio. 2023;22(1):105–31.

[14] Liu ZF, Li ZG, Zhong H, Zeng GM, Liang YS, Chen M, et al. Recent advances in the environmental applications of biosurfactant saponins: a review. J Environ Chem Eng. 2017;5(6):6030–8.

[15] Oleszek WA. Chromatographic determination of plant saponins. J Chromatogr A. 2002;967(1):147–62. 12219927 10.1016/s0021-9673(01)01556-4

[16] Fink R, Filip S. Surface-active natural saponins. Properties, safety, and efficacy. Int J Environ Health Res. 2023;33(7):639–48. 35213278 10.1080/09603123.2022.2043252

[17] Sharma K, Kaur R, Kumar S, Saini RK, Sharma S, Pawde SV, et al. Saponins: a concise review on food related aspects, applications and health implications. Food Chem Adv. 2023;2: 100191.

[18] Jolly A, Hour Y, Lee Y-C. An outlook on the versatility of plant saponins: a review. Fitoterapia. 2024;174: 105858. 38365071 10.1016/j.fitote.2024.105858

[19] Datta D, Talapatra SN, Swarnakar S. Bioactive compounds from marine invertebrates for potential medicines-an overview. Int Lett Nat Sci. 2015;34:42.

[20] D’Auria MV, Paloma LG, Minale L, Riccio R, Debitus C. Structure chacterization by two-dimensional NMR spectroscopy, of two marine triterpene oligoglycosides from a pacific sponge of the genusErylus. Tetrahedron. 1992;48(3):491–8.

[21] Elyakov GB, Stonik VA, Levina EV, Slanke VP, Kuznetsova TA, Levin VS. Glycosides of marine invertebrates—I. A comparative study of the glycoside fractions of pacific sea cucumbers. Comp Biochem Physiol Part B: Comp Biochem. 1973;44(2):325–36.

[22] Kitagawa I, kobayashi M. Saponin and sapogenol. XXVI. Steroidal saponins from the starfish Acanthaster planci L.(Crown of Thorns).(2). Structure of the major saponin Thornasteroside A. Chem Pharm Bull. 1978;26(6):1864–73.

[23] Claereboudt EJS, Caulier G, Decroo C, Colson E, Gerbaux P, Claereboudt MR, et al. Triterpenoids in echinoderms: fundamental differences in diversity and biosynthetic pathways. Mar Drugs. 2019;17(6). 10.3390/md17060352 PMC6627624 31200494

[24] Majinda RR. Extraction and isolation of saponins. Methods Mol Biol. 2012;864:415–26. 22367906 10.1007/978-1-61779-624-1_16

[25] Xiong JL, Lu ZC, Ding N, Ren SM, Li YX. Synthesis of the pentasaccharide moiety of thornasterside A. Eur J Org Chem. 2013;2013(27):6158–66.

[26] Kamyab E, Kellermann MY, Kunzmann A, Schupp PJ. Chemical biodiversity and bioactivities of saponins in Echinodermata with an emphasis on sea cucumbers (Holothuroidea). YOUMARES 9-the oceans: our research, our future. 2020:121. 10.1007/978-3-030-20389-4_7

[27] Bondoc KG, Lee H, Cruz LJ, Lebrilla CB, Juinio-Menez MA. Chemical fingerprinting and phylogenetic mapping of saponin congeners from three tropical holothurian sea cucumbers. Comp Biochem Physiol B Biochem Mol Biol. 2013;166(3–4):182–93. 24036426 10.1016/j.cbpb.2013.09.002

[28] Kalinin VI, Avilov SA, Silchenko AS, Stonik VA. Triterpene glycosides of sea cucumbers (Holothuroidea, Echinodermata) as taxonomic markers. Nat Prod Commun. 2015;10(1):1934578X1501000108. 25920212

[29] Stonik VA, Kicha AA, Malyarenko TV, Ivanchina NV. Asterosaponins: structures, taxonomic distribution, biogenesis and biological activities. Mar Drugs. 2020;18(12). 10.3390/md18120584. 10.3390/md18120584 PMC7760246 33255254

[30] Wahidullah S, Naik BG, Al-Fadhli AA. Chemotaxonomic study of the demosponge Cinachyrella cavernosa (Lamarck). Biochem Syst Ecol. 2015;58:91–6.

[31] Moses T, Papadopoulou KK, Osbourn A. Metabolic and functional diversity of saponins, biosynthetic intermediates and semi-synthetic derivatives. Crit Rev Biochem Mol Biol. 2014;49(6):439–62. 25286183 10.3109/10409238.2014.953628 PMC4266039

[32] Xiong J, Lu Z, Ding N, Ren S, Li Y. Synthesis of the pentasaccharide moiety of thornasterside A. Eur J Org Chem. 2013;2013(27):6158–66.

[33] Juang Y-P, Liang P-H. Biological and pharmacological effects of synthetic saponins. Molecules. 2020;25(21):4974. 33121124 10.3390/molecules25214974 PMC7663351

[34] Baky MH, Elsaid MB, Farag MA. Phytochemical and biological diversity of triterpenoid saponins from family Sapotaceae: a comprehensive review. Phytochemistry. 2022;202: 113345. 35952770 10.1016/j.phytochem.2022.113345

[35] Huang G, Liang J, Chen X, Lin J, Wei J, Huang D, et al. Isolation and identification of chemical constituents from zhideke granules by ultra-performance liquid chromatography coupled with mass spectrometry. J Anal Methods Chem. 2020;2020:8889607. 33457039 10.1155/2020/8889607 PMC7785344

[36] Strege MA. High-performance liquid chromatographic electrospray ionization mass spectrometric analyses for the integration of natural products with modern high-throughput screening. J Chromatogr B. 1999;725(1):67–78. 10.1016/s0378-4347(98)00553-2 10226878

[37] Kind T, Fiehn O. Advances in structure elucidation of small molecules using mass spectrometry. Bioanal Rev. 2010;2(1–4):23–60. 21289855 10.1007/s12566-010-0015-9 PMC3015162

[38] Pyke JS, Callahan DL, Kanojia K, Bowne J, Sahani S, Tull D, et al. A tandem liquid chromatography-mass spectrometry (LC-MS) method for profiling small molecules in complex samples. Metabolomics. 2015;11(6):1552–62.

[39] Kind T, Tsugawa H, Cajka T, Ma Y, Lai Z, Mehta SS, et al. Identification of small molecules using accurate mass MS/MS search. Mass Spectrom Rev. 2018;37(4):513–32. 28436590 10.1002/mas.21535 PMC8106966

[40] Huang FQ, Dong X, Yin X, Fan Y, Fan Y, Mao C, et al. A mass spectrometry database for identification of saponins in plants. J Chromatogr A. 2020;1625: 461296. 32709339 10.1016/j.chroma.2020.461296

[41] Hall MR, Kocot KM, Baughman KW, Fernandez-Valverde SL, Gauthier ME, Hatleberg WL, et al. The crown-of-thorns starfish genome as a guide for biocontrol of this coral reef pest. Nature. 2017;544(7649):231–4. 28379940 10.1038/nature22033

[42] Pratchett MS, Caballes CF, Cvitanovic C, Raymundo ML, Babcock RC, Bonin MC, et al. Knowledge gaps in the biology, ecology, and management of the pacific crown-of-thorns sea star Acanthaster sp. on Australia’s Great Barrier Reef. Biol Bull. 2021;241(3):330–46. 10.1086/717026 35015620

[43] Motti CA, Bose U, Roberts RE, McDougall C, Smith MK, Hall MR, et al. Chemical ecology of chemosensation in Asteroidea: insights towards management strategies of pest species. J Chem Ecol. 2018;44:147–77. 29362949 10.1007/s10886-018-0926-4

[44] Popov RS, Ivanchina NV, Dmitrenok PS. Application of MS-based metabolomic approaches in analysis of starfish and sea cucumber bioactive compounds. Mar Drugs. 2022;20(5):320. 35621972 10.3390/md20050320 PMC9147407

[45] Mendoza-Porras O, Nguyen TV, Shah RM, Thomas-Hall P, Bastin L, Deaker DJ, et al. Biochemical metabolomic profiling of the crown-of-thorns starfish (Acanthaster): new insight into its biology for improved pest management. Sci Total Environ. 2023;861: 160525. 36574554 10.1016/j.scitotenv.2022.160525

[46] Zhao G, Zhao W, Han L, Ding J, Chang Y. Metabolomics analysis of sea cucumber (Apostichopus japonicus) in different geographical origins using UPLC-Q-TOF/MS. Food Chem. 2020;333: 127453. 32659664 10.1016/j.foodchem.2020.127453

[47] Yasumoto T, Hashimoto Y. Properties and sugar components of asterosaponin A isolated from starfish. Agric Biol Chem. 1965;29(9):804–8.

[48] Avilov S, Kalinovskii A, Stonik V. New triterpene glycoside from the holothurian Neothyonidium magnum. Chem Nat Compd. 1990;26:42–5.

[49] Kitagawa I, Kobayashi M, Sugawara T. Saponin and sapogenol. XXV. Steroidal saponins from the starfish Acanthaster planci L. (Crown of Thorns). (1). Structures of two genuine sapogenols, thornasterol A and thornasterol B, and their sulfates. Chem Pharm Bull. 1978;26:1852–63. 10.1248/cpb.26.1852

[50] Kitagawa I, Nishino T, Kobayashi M, Matsuno T, Akutsu H, Kyogoku Y. Marine natural products. VII. Bioactive triterpene-oligoglycosides from the sea cucumber Holothuria leucospilota Brandt (1). Structure of holothurin B. Chemical and Pharmaceutical Bulletin. 1981;29(7):1942–50. 10.1248/cpb.29.1942

[51] Anderson L, Bano S, Bohlin L, Riccio R, Minale L. Studies of Swedish marine organisms. 6. A novel bioactive steroidal glycoside from the starfish Crossaster-papposus. J Chem Res Synopses. 1987;12:366–7.

[52] Ikegami S, Kamiya Y, Tamura S. A new sterol from asterosaponins A and B. Tetrahedron Lett. 1972;13(16):1601–4.

[53] Silchenko AS, Avilov SA, Popov RS, Dmitrenok PS, Chingizova EA, Grebnev BB, et al. Chilensosides E, F, and G-new tetrasulfated triterpene glycosides from the sea cucumber Paracaudina chilensis (Caudinidae, Molpadida): structures, activity, and biogenesis. Mar Drugs. 2023;21(2):114. 36827155 10.3390/md21020114 PMC9964569

[54] Ivanchina NV, Kicha AA, Stonik VA. Steroid glycosides from marine organisms. Steroids. 2011;76(5):425–54. 21194537 10.1016/j.steroids.2010.12.011

[55] Tang HF, Yi YH, Li L, Sun P, Zhang SQ, Zhao YP. Asterosaponins from the starfish Culcita novaeguineae and their bioactivities. Fitoterapia. 2006;77(1):28–34. 16242269 10.1016/j.fitote.2005.07.009

[56] Ha DT, Kicha AA, Kalinovsky AI, Malyarenko TV, Popov RS, Malyarenko OS, et al. Asterosaponins from the tropical starfish Acanthaster planci and their cytotoxic and anticancer activities in vitro. Nat Prod Res. 2021;35(4):548–55. 30887834 10.1080/14786419.2019.1585845

[57] Wang WH, Hong JK, Lee CO, Im KS, Choi JS, Jung JH. Cytotoxic sterols and saponins from the starfish. J Nat Prod. 2004;67(4):584–91. 15104487 10.1021/np030427u

[58] Wang WH, Li F, Alam N, Liu YH, Hong JK, Lee CK, et al. New saponins from the starfish. J Nat Prod. 2002;65(11):1649–56. 12444692 10.1021/np020234r

[59] Wang WH, Li FM, Hong JK, Lee CO, Cho HY, Im KS, et al. Four new saponins from the starfish. Chem Pharm Bull. 2003;51(4):435–9. 10.1248/cpb.51.435 12673001

[60] Kitagawa I, Kobayashi M. On the structure of the major saponin from the starfish Acanthaster planci. Tetrahedron Lett. 1977;18(10):859–62.

[61] Finamore E, Zollo F, Minale L, Yasumoto T. Starfish saponins, part 47. Steroidal glycoside sulfates and polyhydroxysteroids from Aphelasterias japonica. J Nat Prod. 1992;55(6):767–72.

[62] Aswathy M, Banik K, Parama D, Sasikumar P, Harsha C, Joseph AG, et al. Exploring the cytotoxic effects of the extracts and bioactive triterpenoids from Dillenia indica against oral squamous cell carcinoma: a scientific interpretation and validation of indigenous knowledge. ACS Pharmacol Transl Sci. 2021;4(2):834–47. 33860206 10.1021/acsptsci.1c00011 PMC8033758

[63] Kicha AA, Dinh TH, Ivanchina NV, Malyarenko TV, Kalinovsky AI, Popov RS, et al. Three new steroid biglycosides, plancisides A, B, and C, from the starfish Acanthaster planci. Nat Prod Commun. 2014;9(9):1269–74. 25918789

[64] Kicha AA, Kalinovskii AI, Ivanchina NV, Malyarenko TV, Popov RS, Long FK, et al. Minor steroidal triglycoside planciside D from the tropical starfish Acanthaster planci. Chem Nat Compd. 2014;50(6):1032–6.

[65] Pizza C, Pezzulo P, Minale L, Brietmaier E, Pusset J, Tirard P. Starfish saponins 20. Two novel steroidal glycosides from the starfish, Acanthaster planci L. J Chem Res. 1985;5:76–7.

[66] Vien LT, Hanh TTH, Huong PTT, Tu VA, Thanh NV, Lyakhova EG, et al. New steroidal glycosides from the starfish Acanthaster planci. Chem Nat Compd. 2016;52(6):1056–60.

[67] Akbari B, Baghaei-Yazdi N, Bahmaie M, Mahdavi AF. The role of plant-derived natural antioxidants in reduction of oxidative stress. BioFactors. 2022;48(3):611–33. 35229925 10.1002/biof.1831

[68] Halbwirth H. The creation and physiological relevance of divergent hydroxylation patterns in the flavonoid pathway. Int J Mol Sci. 2010;11(2):595–621. 20386656 10.3390/ijms11020595 PMC2852856

[69] Hofstaedter CE, Chandler CE, Met CM, Gillespie JJ, Harro JM, Goodlett DR, et al. Divergent Pseudomonas aeruginosa LpxO enzymes perform site-specific lipid A 2-hydroxylation. mBio. 2024;15(2):e0282323. 10.1128/mbio.02823-23 PMC10865791 38131669

[70] Eeckhaut I, Caulier G, Brasseur L, Flammang P, Gerbaux P, Parmentier E. Effects of holothuroid ichtyotoxic saponins on the gills of free-living fishes and symbiotic pearlfishes. Biol Bull. 2015;228(3):253–65. 26124451 10.1086/BBLv228n3p253

[71] Ikegami S, Kamiya Y, Shirai H. Characterization and action of meiotic maturation inhibitors in starfish ovary. Exp Cell Res. 1976;103(2):233–9. 1033832 10.1016/0014-4827(76)90259-7

[72] Ikegami S, Kamiya Y, Tamura S. Isolation and characterization of spawning inhibitors in ovary of the starfish, Asterias amurensis. Agric Biol Chem. 1972;36(11):2005–11.

[73] Caulier G, Flammang P, Gerbaux P, Eeckhaut I. When a repellent becomes an attractant: harmful saponins are kairomones attracting the symbiotic Harlequin crab. Sci Rep. 2013;3:2639. 24026443 10.1038/srep02639 PMC6505676

[74] Van Dyck S, Caulier G, Todesco M, Gerbaux P, Fournier I, Wisztorski M, et al. The triterpene glycosides of Holothuria forskali: usefulness and efficiency as a chemical defense mechanism against predatory fish. J Exp Biol. 2011;214(Pt 8):1347–56. 21430212 10.1242/jeb.050930

